# Telomere shortening correlates to dysplasia but not to DNA aneuploidy in longstanding ulcerative colitis

**DOI:** 10.1186/1471-230X-14-8

**Published:** 2014-01-09

**Authors:** Mariann Friis-Ottessen, Laila Bendix, Steen Kølvraa, Solveig Norheim-Andersen, Paula M De Angelis, Ole Petter F Clausen

**Affiliations:** 1 Department of Pathology, Division of Diagnostics and Intervention, Oslo University Hospital, Rikshospitalet, Oslo, Norway; 2 Danish Aging Research Center, University of Southern Denmark, Vejle, Denmark; 3 Department of Pathology, Akershus University Hospital, Division of Medicine and Laboratory Sciences, University of Oslo, Oslo, Norway; 4 Department of Pathology, University of Oslo, Oslo, Norway; 5 OUS HF Rikshospitalet, Postboks 4950, Nydalen 0424 Oslo, Norway

**Keywords:** Ulcerative colitis, Ultra-short telomeres, Mean telomere length, DNA-aneuploidy, Dysplasia

## Abstract

**Background:**

Ulcerative colitis (UC) is a chronic, inflammatory bowel disease which may lead to dysplasia and adenocarcinoma in patients when long-lasting. Short telomeres have been reported in mucosal cells of UC patients. Telomeres are repetitive base sequences capping the ends of linear chromosomes, and protect them from erosion and subsequent wrongful recombination and end-to-end joining during cell division. Short telomeres are associated with the development of chromosomal instability and aneuploidy, the latter being risk factors for development of dysplasia and cancer. Specifically, the abrupt shortening of one or more telomeres to a critical length, rather than bulk shortening of telomeres, seems to be associated with chromosomal instability.

**Methods:**

We investigated possible associations between dysplasia, aneuploidy and telomere status in a total of eight lesions from each of ten progressors and four nonprogressors suffering from longstanding UC. We have analyzed mean telomere length by qPCR, as well as the amount of ultra-short telomeres by the Universal STELA method.

**Results:**

An increased amount of ultra-short telomeres, as well as general shortening of mean telomere length are significantly associated with dysplasia in longstanding UC. Furthermore, levels of ultra-short telomeres are also significantly increased in progressors (colons harbouring cancer/dysplasia and/or aneuploidy) compared to nonprogressors (without cancer/dysplasia/aneuploidy), whereas general shortening of telomeres did not show such associations.

**Conclusions:**

Our data suggest that ultra-short telomeres may be more tightly linked to colorectal carcinogenesis through development of dysplasia in UC than general telomere shortening. Telomere status was not seen to associate with DNA aneuploidy.

## Background

Telomeres are specialized structures capping all linear chromosomes, thereby protecting them from erosion during cell division and from wrongful recombination and end-to-end joining [1]. In cancer development telomeres have shown to play a dual role: when cell cycle checkpoints are intact they will act as a tumour suppressor mechanism by bringing about cellular senescence or apoptosis when the telomeres are too short for further division. If however, cell cycle checkpoints are disabled, cells are able to keep dividing past this point and possibly engage in development of chromosomal instability (CIN) [2,3]. It has previously been shown that human cancer cells may harbour ultra-short telomeres [4], and current beliefs are that it is not the shortening of the mean telomere length that might contribute to the development of CIN and altered morphology, but one or a few telomeres shortened to an “ultra-short” state [5,6] These ultra-short telomeres are suspected to form from an abrupt loss of a larger part of a telomere. Oxidative stress is known to cause single strand breaks in DNA [7]. Breaks occurring within the telomeric region of a chromosome are often not repaired, thus leaving the cell with a shortened telomere after genome duplication [8]. Few options for measurement of the amount of ultra-short telomeres are available. Methods for detecting the shorter telomeres, like Q-FISH methods, are generally limited by the need for viable cells, or that they detect the percentage of cells containing short telomeres within a population. They are not able to detect a single, short telomere within a population [9,10]. In 2003, Baird and colleagues presented the single telomere length analysis, STELA, that is able to detect the shortest telomeres in a sample [11]. This analysis was originally limited to the XpYp telomeres, but has since been extended to several autosomal chromosomes as well [12]. This method detects chromosomes where the subtelomeric region is known, as it is dependent upon a chromosome-specific proximal primer. Bendix and colleagues then presented a method in 2010 that provides an estimate of the load of ultra-short telomeres in a DNA sample. This method is a version of the STELA, and was named the Universal-STELA, as it is not limited to chromosomes with known subtelomeric composition [13].

Ulcerative colitis (UC) is an inflammatory bowel disease characterized by inflammation-induced chronic destruction and regeneration of colonic mucosa, resulting in dysplasia and cancer development in some patients. Patients with long lasting UC are at increased risk for development of adenocarcinomas, usually following mucosal transition to dysplasia [14]. It is estimated that 10% of patients suffering from long-term UC will develop preneoplastic changes that might lead to cancer. These patients can be characterized as progressors, as they have preneoplastic changes in one or more locations that are associated with increased risk of progression to invasive cancer. A progressor is typically defined as a patient with dysplasia or cancer. We have also included detection of DNA-aneuploid cell populations as a trait of a progressor colon. A patient not presenting cancer, dysplasia or aneuploid cell populations will thus be considered a nonprogressor using these criteria.

Patients suffering from UC have been shown to harbour shorter telomeres in their colonic mucosa compared to age-matched controls [15-17]. UC-progressors harbour shorter telomeres than do UC-nonprogressors [18] and it has been shown that the mean telomere length of a UC-colon is mainly shortened during the first eight years following onset of the disease [17]. Differences in mean telomere length between low-grade dysplasia (LGD) and high-grade dysplasia (HGD) in the colonic mucosa of patients suffering from long-term UC, with the shortest telomeres reported from mucosa with LGD, have recently been reported [16]. DNA aneuploidy has also been associated with the development of malignancy in UC patients [19-21], and it has been shown that DNA aneuploidy is present in dysplastic as well as non-dysplastic areas of colonic mucosa [21-23]. Thus, both mucosal dysplasia [24,25] as well as DNA aneuploidy [21,26] might predict increased risk for progression to adenocarcinomas in the colon of patients with longstanding UC. Our aim was to examine telomere biology in preneoplastic lesions of patients with longstanding UC. Using the recently developed method, U-STELA, we investigated possible differences in the amount of ultra-short telomeres in aneuploid and diploid dysplastic and non-dysplastic lesions from UC-progressor colectomies. Nonprogressor UC-colons served as controls.

## Methods

### Histopathology

We examined colectomy specimens from 14 patients with longstanding UC from 10–30 years, resected at the Department of Surgery, Rikshospitalet, in the period 1985–1994. The colectomies are part of a patient material that has previously been investigated for aneuploidy-related parameters by our research group [27]. The material included 3 females and 11 males. All but 3 developed UC before the age of 50, and all patients presented extensive colitis.

Use of this material for research purposes has ethical approval from the Regional Ethical Committee, REK S-06062.

10 patients were classified as UC-progressors, i.e. presented with one or more areas of dysplasia or aneuploidy in their colonic mucosa. Tissue specimens for histopathological evaluation were harvested from eight consecutive locations throughout each bowel, in addition to samples collected from gross lesions if present. A total of 78 lesions were available for analysis and fixed in 70% ethanol and embedded in paraffin. Some of the specimens were fixed in formalin only. Tissue sections were cut at 4 μm thickness, stained with hematoxylin-eosin and evaluated independently by two experienced pathologists (OPFC, SNA) according to Riddell et al. [28]. The mucosal morphology was classified independently by each pathologist, and if the evaluation was the same, this diagnosis was used, if not, the lesions were viewed through a double microscope and discussed until consensus was reached. A portion of each fresh tissue specimen was used for isolation of single cells for DNA flow cytometry and for DNA extraction for molecular genetic analysis (see below). Biopsies from 4 colectomies classified as nonprogressor UC-colons were used as controls.

### DNA-flow cytometry

Mucosa adjacent to that used for histopathological examination was mechanically disaggregated in PBS and EDTA using a scalpel. The resulting cell suspensions were filtered through 70 μm nylon mesh and centrifuged at 1500 RPM for 5 minutes. The resulting pellet was resuspended in 1 ml PBS and fixed in 70% ice-cold Ethanol. Samples were stored overnight before preparation for DNA analysis using the procedure of Vindeløv [29], and were analyzed using a FACStar Plus cytometer equipped with 488 nm argon ion laser (BDIS, Spectra Physics, Mountain View, CA, USA). DNA content was assessed based on propidium iodide (PI) fluorescence emission. Aggregates and doublets were excluded from analyses using red fluorescence pulse width of the PI-signal. The purity of epithelial cells in the cell suspensions were analysed by bivariate forward scatter/PI fluorescence [30] and bivariate cytokeratin/PI fluorescence [31] and estimated to be >80%.

### The amount of ultra-short telomeres measured by U-STELA

**Figure 1:**
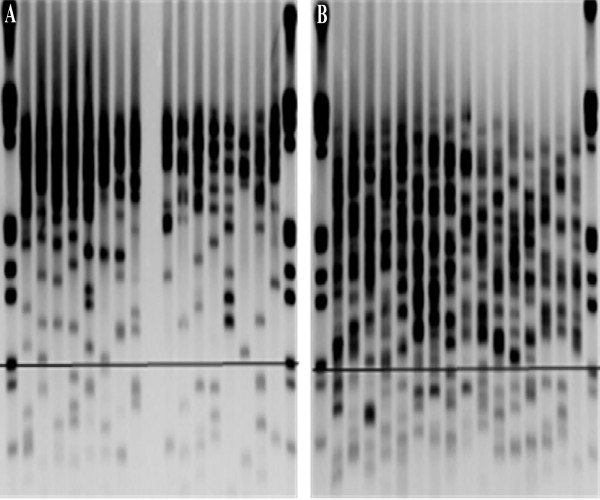
**The Universal STELA.** Excerpts of two typical membranes generated by a Southern blot, visualizing the ultra-short telomeres as analysed by the Universal STELA. Each lane represents a PCR-reaction. Eight separate PCR-reactions are performed for each lesion analysed. The horizontal bar marks 900 basepairs. Bands below this bar represent single telomeres and are counted for the analyses. **A)** Two lesions from a nonprogressor. **B)** Two lesions from a progressor.

The Universal STELA method was performed as described by Bendix and colleagues [13], with a few minor alterations. Fifty ng DNA, extracted from each of the 78 lesions, was digested using a 1:1 mix of restriction enzymes NdeI and MseI. Ten ng of digested DNA was mixed with 50 μmol of the oligoes 42-mer and 11 + 2-mer. The mix was ramped from 65°C to 16°C over one hour, before adding 20U T4 ligase together with 1x NEB buffer 2 and 1x ATP (NEB). This mixture was left at 16°C for 12 hours before 20 U t4 ligase together with 10^-3^ μM telorette 3, 1x ATP and 1x NEB buffer 2 was added (to a total volume of 25 μl). The mixture was kept for 12 hours at 35°C, followed by a 20 minutes inactivating step at 65°C. PCR was then performed in a 12 μl reaction containing 40 pg template, 1x Failsafe PCR premix H (Epicentre), 0, 1 μM of each of primers Teltail and Adapter, and 1,25U Failsafe Enzyme mix (Epicentre). The reactions were performed on a 2720 Thermal Cycler (Applied Biosystems) with the conditions one cycle of 68°C for 5 minutes and 95°C for 2 minutes, followed by 24 cycles of 95°C for 15 seconds, 58°C for 20 seconds and 72°C for 11 minutes, and one cycle of 72°C for 15 minutes. The PCR-products were separated on a 0, 85% TBE Seakem agarose gel at 5 V/cm, and transferred to a positively charged nylon membrane by Southern blotting using a vacuum blotter. The blotted DNA was then hybridized overnight to a digoxigenin (DIG)-labelled probe specific for the telomeric sequence and further incubated with a DIG-specific antibody coupled to alkaline phosphatase (Roche). The probe was visualised by CDP-Star, a chemiluminescent substrate (Roche) and detected using a Kodak Image Station 4000R. The fragment sizes of the PCR-products are determined based on a DIG-labelled molecular weight marker (Roche), using Molecular Imaging from Carestream. A cut-off was set at 900 bp, as normal colonic mucosa had very few bands using this threshold (data not shown), and in UC-samples the bands were presented as single, separated bands. Bands were counted manually. An illustration of U-STELA results is shown in Figure 1.

### Mean telomere length analysis

Telomere mean length quantification was carried out with an adaptation of the Q-PCR methods described by Cawthon [32,33]. TaqMan Copy Number Reference Assay RNase P (located at 14q11.2 ) or alternatively TaqMan Copy Number Reference Assay Tert (located on 5p5.33 ) (Both Applied Biosystems, Denmark) was run at recommended conditions to measure the single copy gene copy number (SCG). For measurement of telomere repeat copies (T) primers telg – 5′-ACA CTA AGG TTT GGG TTT GGG TTT GGG TTT GGG TTA GTG T-3′ and telc – 5′-TGT TAG GTA TCC CTA TCC CTA TCC CTA TCC CTA TCC CTA ACA-3′, [32] were added at a concentration of 0.5 μM to Ssofast EvaGreen Supermix with low ROX (BioRad, Denmark). Cycling conditions were: 2 min at 50°C, 2 min of 95°C, followed by 2 cycles of 95°C for 15 s, 52°C for 15 s and 36 cycles of 95°C for 15 s, 62°C for 15 s and 71°C for 15 s. The PCR was run on a 7900HT Fast Real-Time PCR System, as presented by Bendix and colleagues [34]. Following amplification, telomeric to single copy gene (T/SCG) ratios were derived from a standard curve using the 7900HT Sequence Detection System version SDS2.3 (Applied Biosystems, Denmark). The results were presented as T/SCG ratios. Samples were run twice in triplicates and the mean of these runs was used. Average coefficient of variance (CV) for T/SCG using RNAse P was 10.5%. Average CV for T/SCG using TERT was 11.3%.

We have used two different primer sets for SCG because the mean length is derived from the ratio between telomere product and single copy gene-product, and therefore there is a potential risk that we will either over- or underestimate the telomere length of the lesions comprised of aneuploid cells if the chosen SCG should be duplicated or deleted in these cells. TERT was chosen to supplement the RNAseP. TERT is located on chromosome 5p, a region often amplified in aneuploid, sporadic colorectal cancers [35] as well as in aneuploid UC [36]. By comparing the T/SCG-ratios derived from the aneuploid lesions alone, we note correlation in all but six lesions, four of which are adenocarcinomas (Additional file 1). To further overcome this potential limitation we repeated statistical analysis with only diploid lesions (presented in Additional file 2). Based on these results RNAse P was selected as SCG for our analyses.

### Statistics

The relationships between mean telomere length/amounts of ultra short telomeres and morphological changes/DNA-ploidy-status were evaluated using non-parametric t-tests and one-way ANOVA and the multivariable, general linear model (GLM). To address the possible differences generated by natural variation between patients we also utilized a mixed model analysis that took into account that each patient generated multiple lesions. Tests were performed in PASW statistics 18 (Chicago, Illinois) and in GraphPad Prism (San Diego, California). All tests were two-sided and a p-level of 0.05 denoted significance.

P-values from t-tests, including the Universal-STELA and both SCG primers tested for measuring mean telomere length are listed in Additional file 2.

## Results

### Histopathology

From the analyzed 14 colectomies 10 colons revealed at least one area with dysplasia, adenocarcinoma or DNA aneuploidy out of the eight areas analyzed (see Table 1). Within the same lesion the mucosal morphology might differ; in such cases the highest degree of abnormality was registered. Of the 78 areas analyzed, 40 areas did not show dysplasia (51.3%), 15 areas were considered indefinite for dysplasia (19.2%), 17 areas had either low or high grade dysplasia (21.8%) and 6 adenocarcinomas (7.7%) were found. These ten colectomy specimens were therefore considered progressors, including one bowel with 4 areas indefinite for dysplasia and four non-dysplastic areas. Two lesions were not available for analyses. Four colectomies presented only diploid, non-dysplastic lesions. These were classified as nonprogressors and used as controls.

**Table 1:** UC-patient materials

**Sample ID**	**Aneuploid areas**	**Dysplasia**				**Adeno-carcinoma**	**Universal STELA**	**qPCR**
		**No**	**Indefinite**	**Low-grade**	**High-grade**			
**Nonprogressors**								
**S9**	0	8	0	0	0	0	X	X
**S48**	0	8	0	0	0	0	X	X
**S62**	0	8	0	0	0	0	X	X
**S216**	0	7	0	0	0	0	X	
**Total**		**31**						
**Progressors**								
**S30**	1	7	0	0	0	1	X	
**S70**	0	6	0	0	1	1	X	X
**S99**	2	0	2	2	3	1		X
**S132**	3	1	5	2	0	0	X	X
**S159**	3	2	0	3	1	0		X
**S169**	6	1	1	2	1	3	X	X
**S191**	3	6	1	1	0	0		X
**S199**	1	5	1	1	0	0	X	X
**C1514**	2	7	1	0	0	0	X	X
**C1729**	0	4	4	0	0	0	X	X
**Total**	**21**	**39**	**15**	**11**	**6**	**6**		

Due to limited amounts of DNA from some colectomy-samples only one of the methods could be performed on some of the cases (see Table 2).

**Table 2:** Lesions available for telomere analyses

	**Aneuploid lesions**		**Diploid lesions**	
	**U-STELA**	**qPCR**	**U-STELA**	**qPCR**
**Nonprogressor lesions**				
**Non-dysplasia**	0	0	27	22
**Dysplasia**	0	0	0	0
**Total**	0	0	27	22
**Progressor lesions**				
**Non-dysplasia**	4	7	24	25
**Dysplasia**	8	13	15	24
**Total**	12	20	39	49

### DNA flow cytometry

The DNA ploidy status of the colectomies has been previously assessed [27]. Within the 10 progressor colons analyzed 57 lesions were diploid (70%) and 21 (30%) lesions harboured aneuploid populations (see Table 1). In addition one diploid and five aneuploid adenocarcinomas were recorded. Two lesions were not available for analyses. We detected both aneuploid and diploid non-dysplastic areas as well as aneuploid and diploid dysplastic areas. One colon was diploid all through, but presented multiple sites of dysplasia. All nonprogressor lesions were diploid.

The proportion of aneuploid cells varied from around 5% to 10% within the lesions presenting aneuploid cell populations, with exception of one case (S169) presenting aneuploid cell counts estimated to be between 15 and 50% and one cancer (S99) represented only in qPCR.

### Ultra-short telomeres and associations

**Figure 2:**
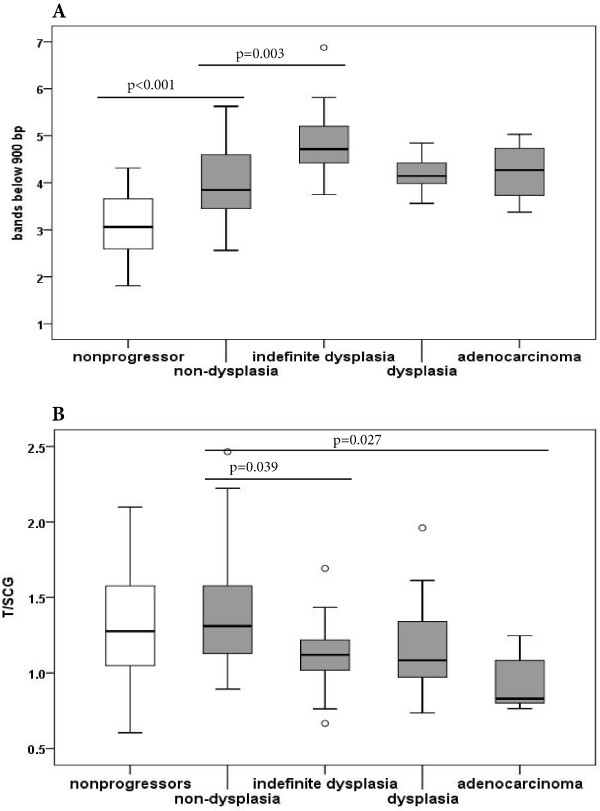
**Ultra-short telomeres and mean telomere length in nonprogressors and progressors. A)** The amount of ultra short telomeres differs significantly from nonprogressors to progressors (p < 0.001). Within progressors there is a significant rise in the amount of ultra short telomeres in lesions indefinite for dysplasia compared to non-dysplastic lesions. No difference is seen within the different degrees of dysplasia and adenocarcinoma. **B)** No significant difference was detected in mean telomere length from nonprogressors to progressors. Within the progressors we noted a significant shortening in mean telomere length in lesions indefinite for dysplasia and in adenocarcinomas from non-dysplastic lesions. (ANOVA with Bonferroni post hoc test).

Overall we found a significant difference in the amount of ultra short telomeres between progressor and nonprogressor colons (p < 0.001) (Figure 2A). This significance holds also when comparisons are restricted to diploid, non-dysplastic lesions of the progressors (see Additional file 2).

Within the progressors we defined four morphological parameters; non-dysplasia, indefinite for dysplasia, dysplasia and adenocarcinoma. Dysplasia comprises both high-grade and low-grade dysplasia, due to few dysplastic lesions and no statistical significant difference in the amount of ultra short telomeres detected between the two groups (p = 0.534).

Dividing the dysplastic lesions as mentioned previously, we noted a significant increase in the amount of ultra-short telomeres in areas indefinite for dysplasia when compared to non-dysplastic lesions (p = 0.003). The amount of ultra short telomeres detected in lesions indefinite for dysplasia did not differ significantly from amounts detected in dysplastic lesions or to the adenocarcinomas (ANOVA with Bonferroni post hoc test) (Figure 2A). No significant difference in the levels of ultra short telomeres was detected between lesions with and without aneuploid populations. Within the diploid lesions of the progressors we found significantly raised levels of ultra short telomeres in the dysplastic lesions compared to the non-dysplastic lesions (p = 0.014), whereas no significant difference was detected between the aneuploid dysplastic areas compared to aneuploid non-dysplastic areas, and diploid dysplasia did not differ from aneuploid dysplasia.

Significant differences related to the location within the colon were not observed. Within the progressor colons we detected a trend towards elevated number of ultra short telomeres in the dysplastic lesions (indefinite for dysplasia and/or dysplasia) compared to the non-dysplastic lesions of the same colon. No such trend was detected when comparing diploid and aneuploid lesions with respect to the amount of ultra short telomeres, within a colon.

To further test if the amount of ultra short telomeres can be an independent predictor of dysplasia in progressor colons we built a model that included morphology, DNA ploidy status, duration of disease combined with onset of UC and location of the biopsy within the colon. This was tested using a multivariable, general linear model (multivariable GLM). The results of this test supported our findings of ultra short telomeres being a possible predictor of dysplasia in progressors (p = 0.007) even when the other parameters are taken into account.

As all colectomies contributed eight lesions to our analyses we also performed a mixed model analysis that considers the natural variation between each patient. Still we found a significant difference between progressor and nonprogressor cases (p = 0.014). Within the ten progressor colectomies we found a significant difference between the different degrees of dysplasia when LGD, HGD and adenocarcinomas were combined (p = 0.033). No significant difference was seen comparing diploid and aneuploid lesions.

Other parameters tested were patients’ age at onset of disease, and duration of disease, as our patient material varied greatly with respect to both parameters. No association between the amount of ultra-short telomeres and either of these parameters was seen (data not shown). Gender did not associate with the amount of ultra-short telomeres.

### Mean telomere length and associations

In this analysis we did not find any difference in mean telomere length between progressors and nonprogressors, neither in the whole material, nor when analysing only non-dysplastic, diploid lesions (Figure 2B and Additional file 2).

Within the progressors a significant shortening in mean telomere length was seen when comparing a group comprising lesions indefinite for dysplasia and dysplasia, to non-dysplastic lesions of the progressors-colons (p = 0.002) (see Additional file 2).

This remained significant also when restricting the analysis to diploid lesions alone (p = 0.008). No significant difference in mean telomere length was found comparing indefinite for dysplasia, dysplasia and adenocarcinoma, but we noted a significant reduction in mean telomere length for lesions indefinite for dysplasia as well as for adenocarcinomas compared to the non-dysplastic lesions of the progressors (ANOVA p = 0.003; Bonferroni post hoc test, p = 0.039 and p = 0.027, respectively) (Figure 2B).

Between the segments of the colectomies we found within the progressors a significant reduction of mean telomere length in the transverse colon compared to the ascending colon (ANOVA, Bonferroni post hoc test, p = 0.011). The descending part of the colectomies did not differ from either the transverse or the ascending colon.

No significant difference was detected between the non-dysplastic and dysplastic aneuploid lesions, or between diploid and aneuploid dysplasia. When comparing different lesions within each progressor colon we detected a trend towards reduced mean telomere length in dysplastic versus non-dysplastic lesions within colons. No such trend was seen between diploid and aneuploid lesions of the colons.

We repeated the calculations with the mean length normalized to TERT. The findings were overall similar to those registered when normalizing to RNaseP, but difference between progressors/nonprogressors was now borderline significant (p = 0.08), and the difference in mean telomere length between diploid and aneuploid lesions within the progressors was statistically significant (p = 0.04) (see Additional file 2).

In a multivariable GLM where we tested for the effect of biopsy location, degree of dysplasia, DNA ploidy status and age at onset combined with duration of UC, we found that in the progressor colectomies, both location of the biopsy (p =0.006) and degree of dysplasia (p = 0.012) might be individually influential to the mean telomere length.

Since each colectomy provided several lesions we performed a GLM considering the patient variations. No significant difference was found when comparing progressor colectomies to nonprogressor colectomies. Within the progressor cases there was a significantly longer mean telomere length in non-dysplastic compared to dysplastic lesions when dysplastic lesions contained all dysplasia (indefinite, LGD, HGD and adenocarcinoma) (p = 0.028). The significance disappeared when lesions indefinite for dysplasia was viewed as a separate group.

Mean telomere length did not associate with gender, patients’ age at disease onset or to duration of UC.

## Discussion

It has been suggested that it is not necessarily the general shortening of telomeres that causes chromosomal instability and aneuploidy, but rather one or more critically short telomeres [5]. We have examined the amount of ultra-short telomeres [13] in the colonic mucosal cells of patients suffering from long standing UC, who have developed dysplasia and/or DNA-aneuploidy. We compared these findings with those from patients suffering from long standing UC without displaying dysplasia or DNA-aneuploidy. Patients with long standing UC have previously been reported to exhibit shortening of mean telomere length in their colonic mucosa, but the amount of ultra-short telomeres has so far not been investigated.

One major finding is a highly significant increase in number of ultra-short telomeres in progressor colons compared to nonprogressor colons, both when including all lesions of the progressors as well as when restricting the progressor lesions to non-dysplastic, diploid lesions only. Furthermore, both simple pair-wise comparisons and multivariate analysis showed that the degree of increase in ultra-short telomeres associated much better with degree of dysplasia than with ploidy status, disease duration or location. This was seen whether we included the adenocarcinomas in the dysplasias, or not. In contrast; there was no difference in mean telomere length between nonprogressors and progressors, although we also here found an association of mean telomere length with dysplasia. The fact that the number of ultra-short telomeres was significantly higher in dysplastic areas compared to non-dysplastic areas suggests that the occurrence of ultra-short telomeres is an early event in the progression towards dysplasia and invasive cancer. This is further supported by the fact that the lesions defined as indefinite for dysplasia, which applies to epithelial changes that exceed the limit for regeneration but are insufficient for an unequivocal diagnosis of dysplasia [28], have significantly more ultra-short telomeres than non-dysplastic mucosa. Elevated numbers of ultra-short telomeres in dysplastic compared to nondysplastic areas were also seen comparing dysplastic to the non-dysplastic lesions using a mixed model analysis. The mixed model analysis compensates for inter-patient differences, and gives a strong indication that the differences seen are indeed caused by differences in the mucosal morphology. Differences in the number of ultra-short telomeres were not seen between diploid and aneuploid lesions within the examined colectomies, when examined by the mixed model analysis.

Thus; increased numbers of ultra-short telomeres may be a marker of UC-colons harbouring dysplasia, cancer and/or aneuploidy, but may also just predict the future development of these conditions.

Short telomeres predispose to end-to-end fusions of chromatids, ultimately leading to chromosome breaks and chromosome losses [18,37]. We did not find any associations between the amount of ultra-short telomeres and aneuploidy. However; it has been previously shown, and confirmed by our results, that not all dysplasias are aneuploid. Aneuploidy develops in non-dysplastic as well as dysplastic mucosa [19,23], and associations between dysplasia and ploidy status respectively, to telomere shortening, may be different. We did not detect any statistically significant difference when comparing aneuploid progressor lesions to diploid progressor lesions, and diploid dysplasia did not differ from aneuploid dysplasia, further indicating that DNA aneuploidy is not strongly associated with elevated levels of ultra-short telomeres. However, we have analysed areas of UC-colons that had developed DNA-aneuploidy, and most lesions included a rather low amount of aneuploid cells (see Result section; DNA flow cytometry for details). We can therefore not exclude an association between aneuploid cells and ultra-short telomeres. To investigate this problem, pure populations of sorted aneuploid cells should be analyzed.

We find that dysplastic lesions in longstanding UC have significantly shorter mean telomere length compared to non-dysplastic areas, when we combine lesions indefinite for dysplasia and dysplastic lesions. This holds true also when separating lesions indefinite for dysplasia from dysplastic lesions: both parameters had significantly reduced mean telomere length compared to non-dysplastic lesions, but no difference was seen between lesions indefinite for dysplasia and the dysplastic lesions. This was seen whether we included adenocarcinomas in the dysplastic lesions or excluded them from the data completely, but not when adenocarcinoma was also included in the statistics as a separate group. The adenocarcinomas presented significantly shorter mean telomere length than seen in non-dysplastic lesions, whereas both indefinite for dysplasia and dysplastic lesion had no statistical significant difference in mean telomere length from non-dysplastic lesions. These results indicate a gradual shortening from areas indefinite for dysplasia through dysplasia to adenocarcinoma. Considered together with the results from the amount of ultra-short telomeres discussed above, our results seem to illustrate damage to telomeres by oxidative stress in the colonic mucosa of UC-patients, as oxidative stress may cause shortening of mean telomere length as well as an abrupt shortening of a single or of a few telomeres.

Shortening of mean telomere length in non-dysplastic mucosa of UC patients has previously been shown, and was also reported to be associated with chromosomal instability [18]. Shortened mean telomere length of the colonic mucosa has also been reported in association with development of colorectal cancer in patients suffering from UC [16,17]. Other parameters that have been found to associate with such development are morphological changes and development of DNA-aneuploidy [16,17,19,21,26,37,38]. Furthermore, it has been reported that patients suffering from UC have generally shorter mean telomere length in their colonic mucosa compared to non-UC control patients. The location of the biopsies within the bowel did not affect these results, and there are indications that the main shortening of telomeric length occurred during the initial eight years of disease [15-18]. It has also been shown by others that progressors have generally shorter telomeres than registered in nonprogressors [18]. The patients in the present study have suffered from UC from a minimum of 10 years, some as long as 30 years, and the mean telomere length in their colonic mucosa may therefore be reduced too much to detect possible differences between nonprogressors and progressors. This may be an explanation to the finding of no significant difference in mean telomere length between progressors and nonprogressors in our study. Another possible explanation might be that we have a rather small number of patients coupled with a rather high coefficient of variation for mean telomere length measurement with either of the single control genes used (10.5% and 11.3%).

In a recent report it was shown that patients developing UC after the age of 50 present longer telomeres than patients developing UC at an earlier age [39]. In our material only two progressor cases and one nonprogressor case had reached the age of 50 before reporting signs of UC, and none of them differed from the rest of the progressor cases with respect to mean telomere length or to the amount of ultra-short telomeres. A possible explanation for the lack of differences detected between these groups might also be severity of disease, as all patients included in our study had developed pancolitis before colectomy.

The difference we noted in the two SCGs used to estimate mean telomere length might be caused by amplification of chromosome arm 5p, the arm that contains the h-TERT locus. Amplification of 5p is often seen both in aneuploid, sporadic colorectal cancers [35] and even more frequently in aneuploid UC [36]. Duplication of h-TERT in some of the aneuploid lesions can report a too low T/SCG value in these samples, and lower the mean value of the combined aneuploid populations. This will again produce a bigger difference in mean telomere length between the diploid and aneuploid populations, than what is actually the case, and ultimately lead to a false statistical significance. We therefore considered RNaseP to be a more accurate SCG for this analysis.

Expression of hTERT is considered the limiting factor of telomerase assembly, and thus of telomerase activity. The presence of hTERT elevation in UC with mild inflammation [40] could perhaps be a protective agent against development of ultra-short telomeres within nonprogressors.

An important finding in the present communication is that the telomere related abnormalities in mucosa from patients with long-standing UC are much less significant when measuring mean telomere length than when measuring number of ultra-short telomeres. This finding suggests that the mechanism behind telomere pathology is not only increased replication due to cell renewal, since this would have resulted in a decrease in mean telomere length. In contrast it has been shown in a model system [41], that stochastic telomere breaks resulting in an increase in number of ultra-short telomeres without concomitant decrease in mean telomere length can be the result of severe oxidative damage to telomeres. Our findings could suggest that oxidative damage is a major reason for telomere pathology in UC mucosa, a hypothesis that is supported by the finding of elevated levels of oxidative stress in the mucosa of UC patients [42,43].

To what extend the high frequency of ultra-short telomeres in colon mucosa cells of patients with UC have causal relationship with increased risk of UC-related colon cancer in these patients is still unknown. A carcinogenic pathway from short telomeres linked to breakage-fusion-bridge cycles to development of chromosome aberrations and aneuploidy, and ultimately to malignant transformation, has been indicated previously in a model system [44].

## Conclusion

We have confirmed that mean telomere length is reduced in dysplastic colonic mucosa in progressor colons of longstanding UC compared to non-dysplastic mucosa, and shown that the amount of ultra-short telomeres is increased in the same situation. Furthermore, we have shown that the amount of ultra-short telomeres is significantly increased in colonic mucosa of progressors compared to nonprogressors, whereas such a difference was not found with respect to mean telomere length. This suggests that ultra-short telomeres are a more sensitive marker of tumour progression than mean telomere length in longstanding UC, and also that the Universal-STELA may be a more accurate estimator of a progressor than qPCR and mean telomere length measurement.

## Competing interests

This work was supported by the South-Eastern Norway Regional Health Authority and by Stiftelsen UNI. These organisations had no role in the collection, analyses or interpretations of the data or in writing the report.

## Authors’ contributions

MFO performed the STELA-analyses, organized the databases, did the statistical analyses and wrote the paper. LB performed the qPCR-analyses and provided critical insights for the writing of the paper. PMD was involved in planning and organizing the project. OPC is the principal investigator, supervisor and guarantor of the study. SNA provided the patient material for the study. SK and the other authors were all involved in manuscript preparation, evaluation, editing and revision. All authors read and approved the final manuscript.

## Pre-publication history

The pre-publication history for this paper can be accessed here:

http://www.biomedcentral.com/1471-230X/14/8/prepub

## Supplementary Material

Additional file 1 **Mean telomere length estimated with two different single copy genes (SCG).** The relation between mean telomere estimated with the regular SCG RNAseP (TS_RNAseP) and the mean telomere length with the alternative TERT gene (TS_TERT). There is correlation in all samples but six (marked with arrows). These six samples are from progressors and all are aneuploid. Of the six lesions four were adenocarcinomas, one was dysplastic and one was a non-dysplastic lesion. Click here for file

Additional file 2 **Mean and p-values from t-tests. **Mean values and p-values of t-tests comparing progressors to nonprogressors, and comparing the different parameters from the progressor colons. Results are reported for U-STELA and for two different mean telomere analyses. One was using RNAseP as SCG, the other using TERT. Click here for file
