# Supplementary figures and images for: Telomere shortening correlates to dysplasia but not to DNA aneuploidy in longstanding ulcerative colitis
Source: BMC Gastroenterol. 2014 Jan 9;14:8. doi: 10.1186/1471-230X-14-8 (PMC3893461; doi:10.1186/1471-230X-14-8)

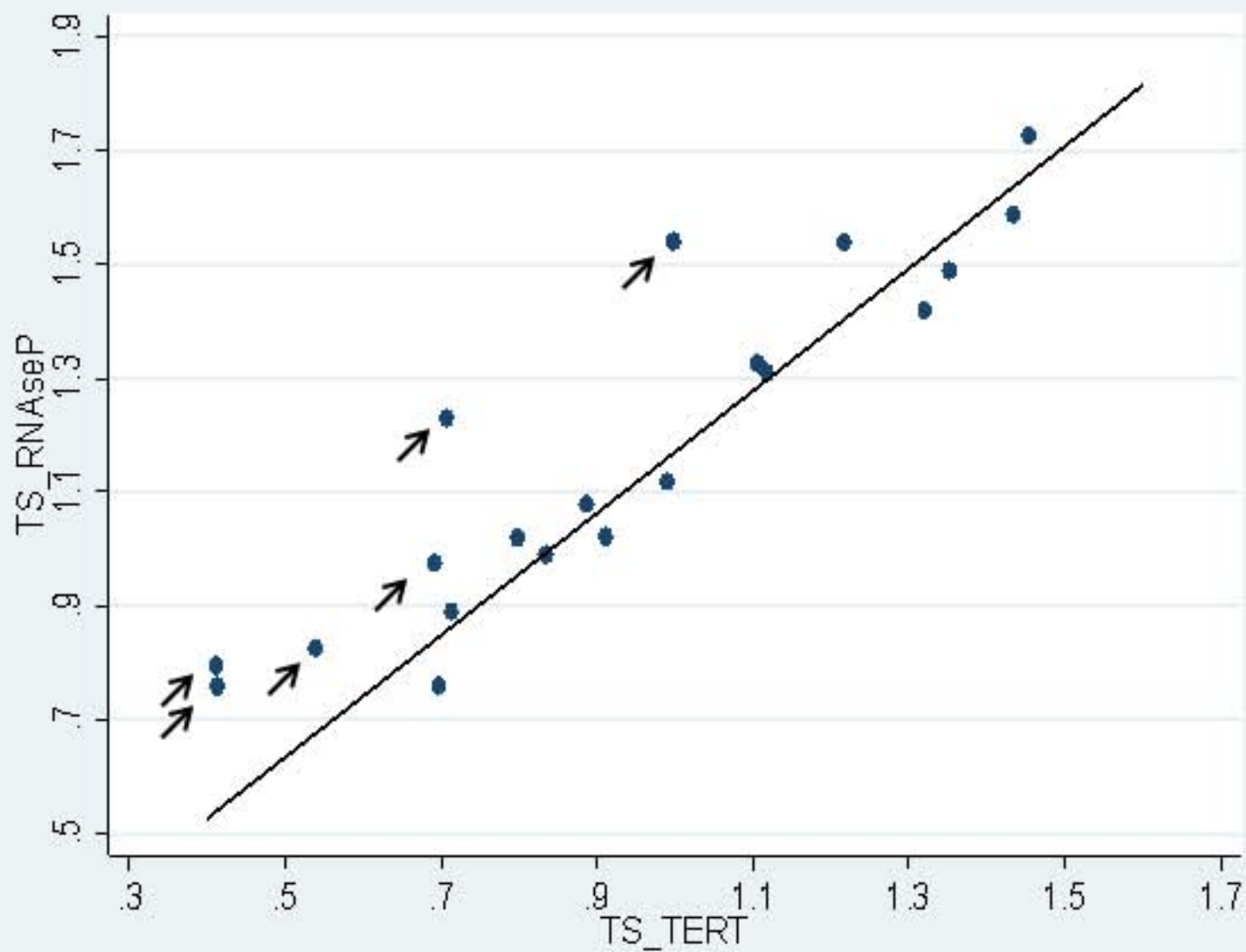

Supplement: Additional file 1 — Mean telomere length estimated with two different single copy genes (SCG). The relation between mean telomere estimated with the regular SCG RNAseP (TS_RNAseP) and the mean telomere length with the alternative TERT gene (TS_TERT). There is correlation in all samples but six (marked with arrows). These six samples are from progressors and all are aneuploid. Of the six lesions four were adenocarcinomas, one was dysplastic and one was a non-dysplastic lesion. [file 1471-230X-14-8-S1.pdf]
