# Supplementary material for: Telomere shortening correlates to dysplasia but not to DNA aneuploidy in longstanding ulcerative colitis
Source: BMC Gastroenterol. 2014 Jan 9;14:8. doi: 10.1186/1471-230X-14-8 (PMC3893461; doi:10.1186/1471-230X-14-8)
Supplement: Additional file 2 — Mean and p-values from t-tests. Mean values and p-values of t-tests comparing progressors to nonprogressors, and comparing the different parameters from the progressor colons. Results are reported for U-STELA and for two different mean telomere analyses. One was using RNAseP as SCG, the other using TERT. [file 1471-230X-14-8-S2.pdf]

Supplementary table 1:

		U-STELA		Mean (RNAseP)		Mean (TERT)	
TOTAL UC COLECTOMIES		Progressors vs. non-progressors		Progressors vs. non-progressors		Progressors vs. non-progressors	
	N	51	27	69	22	67	22
	Mean	4.24	3.06	1.26	1.31	1.11	1.29
	p	<0.001		0.56		0.08	
		Non-dysplastic, diploid progressor lesions vs nonprogressors					
		Progressors vs. non-progressors		Progressors vs. non-progressors		Progressors vs. non-progressors	
	N	24	27	25	22	24	22
	Mean	3.96	3.06	1.43	1.31	1.29	1.26
	p	<0.001		0.35		0.83	
PROGRESSOR COLECTOMIES		Diploid vs. aneuploid		Diploid vs. aneuploid		Diploid vs. aneuploid	
	N	39	12	49	20	47	20
	Mean	4.19	4.39	1.29	1.18	1.13	0.93
	p	0.46		0.27		0.04	
		Non-dysplasia vs. Dysplasia*		Non-dysplasia vs. dysplasia*		Non-dysplasia vs. Dysplasia*	
	N	28	23	32	37	31	36
	Mean	3.97	4.57	1.41	1.11	1.22	0.93
	p	0.007		<0.001		<0.001	
		Only diploid lesions					
		Non-dysplasia vs. Dysplasia*		Non-dysplasia vs. Dysplasia*		Non-dysplasia vs. Dysplasia*	
	N	24	15	25	24	24	23
	Mean	3.96	4.57	1.43	1.14	1.26	0.99
	p	0.03		0.007		0.008	

*dysplasia includes indefinite for dysplasia, LGD, HGD and adecocarcinoma
